# SARS-CoV-2 spike glycosylation affects function and neutralization sensitivity

**DOI:** 10.1128/mbio.01672-23

**Published:** 2024-01-09

**Authors:** Fengwen Zhang, Fabian Schmidt, Frauke Muecksch, Zijun Wang, Anna Gazumyan, Michel C. Nussenzweig, Christian Gaebler, Marina Caskey, Theodora Hatziioannou, Paul D. Bieniasz

**Affiliations:** 1 Laboratory of Retrovirology, The Rockefeller University, New York, New York, USA; 2 Laboratory of Molecular Immunology, The Rockefeller University, New York, New York, USA; 3 Howard Hughes Medical Institute, The Rockefeller University, New York, New York, USA; Dana-Farber Cancer Institute, Boston, Massachusetts, USA

**Keywords:** SARS-CoV-2, antibody function, neutralization, glycosylation

## Abstract

The glycosylation of viral envelope proteins can play important roles in virus biology and immune evasion. The spike (S) glycoprotein of severe acute respiratory syndrome coronavirus-2 (SARS-CoV-2) includes 22 N-linked glycosylation sequons and 17 O-linked glycosites. We investigated the effect of individual glycosylation sites on SARS-CoV-2 S function in pseudotyped virus infection assays and on sensitivity to monoclonal and polyclonal neutralizing antibodies. In most cases, the removal of individual glycosylation sites decreased the infectiousness of the pseudotyped virus. For glycosylation mutants in the N-terminal domain and the receptor-binding domain (RBD), reduction in pseudotype infectivity was predicted by a commensurate reduction in the level of virion-incorporated S protein and reduced S trafficking to the cell surface. Notably, the presence of a glycan at position N343 within the RBD had diverse effects on neutralization by RBD-specific monoclonal antibodies cloned from convalescent individuals. The N343 glycan reduced the overall sensitivity to polyclonal antibodies in plasma from COVID-19 convalescent individuals, suggesting a role for SARS-CoV-2 S glycosylation in immune evasion. However, vaccination of convalescent individuals produced neutralizing activity that was resilient to the inhibitory effect of the N343 glycan.

**IMPORTANCE:**

The attachment of glycans to the spike proteins of viruses during their synthesis and movement through the secretory pathway can affect their properties. This study shows that the glycans attached to the severe acute respiratory syndrome coronavirus-2 spike protein enable its movement to the cell surface and incorporation into virus particles. Certain glycans, including one that is attached to asparagine 343 in the receptor-binding domain of the spike protein, can also affect virus neutralization by antibodies. This glycan can increase or decrease sensitivity to individual antibodies, likely through direct effects on antibody epitopes and modulation of spike conformation. However, the overall effect of the glycan in the context of the polyclonal mixture of antibodies in convalescent serum is to reduce neutralization sensitivity. Overall, this study highlights the complex effects of glycosylation on spike protein function and immune evasion.

## INTRODUCTION

Severe acute respiratory syndrome coronavirus-2 (SARS-CoV-2) is the causative agent of COVID-19 disease and has caused a devastating pandemic (1, 2). SARS-CoV-2 encodes a spike (S) glycoprotein, which binds angiotensin-converting enzyme 2 (ACE2) and mediates viral entry into host cells (3–6). The 1,273 amino acid (aa) S glycoprotein consists of a signal peptide followed by the S1 subunit (aa 13–685) and the S2 subunit (aa 686–1,273). These two subunits are separated by a furin cleavage site (PRRAR), abrogation of which can increase virus infectivity in some circumstances (7). The receptor-binding domain (RBD, aa 319–541) that is responsible for ACE2 binding (6) and the N-terminal domain (NTD), both encoded within S1, are the major targets of neutralizing antibodies. Like other viral envelope glycoproteins, including human immunodeficiency virus-1 (HIV-1) (8), SARS-CoV-2 S protein is heavily glycosylated (9–11), and approximately 40% of the protein surface is shielded by glycans (12). The majority of this shield is comprised of N-linked oligomannose-type or complex glycans, linked to 22 sites (Asn-X-Ser/Thr) on the S protein (13). Additionally, 17 O-linked glycosites have been identified by biochemical methods (14–16).

Glycosylation of viral envelope proteins can play an important role in virus–host interactions (17). In the case of HIV-1, for example, N-linked glycans are essential for correct folding and processing of gp120 as well as structural rearrangements required for receptor binding (18). The HIV-1 glycan shield also plays a crucial role in preventing neutralizing antibodies from binding to gp120 (19). Likewise, S glycosylation affects SARS-CoV-2 infection (20). Blocking N-glycan biosynthesis onto SARS-CoV-2 S protein and, to a lesser extent, O-glycan elaboration reduces viral infectivity (21). Additionally, cryo-electron microscopy studies have revealed that the N-glycan at position N343 in the RBD facilitates the transition of the S protein to the “open” conformation, which is important for ACE2 binding (22). Accordingly, mutation of this site (N343Q) reduced viral entry into ACE2-expressing cells (23).

Little is known about how SARS-CoV-2 S glycosylation might affect immune surveillance. It is conceivable that glycans sterically shield underlying epitopes from recognition by antibodies, as is the case in HIV-1 (19). Many SARS-CoV-2 neutralizing antibodies target the RBD and can be divided into four broad classes based on the epitopes that are targeted (24–28). Class 1 antibodies recognize epitopes overlapping the ACE2-binding site and bind only to “up” RBDs. Class 2 antibodies bind both “up” and “down” RBDs and also block ACE2 binding. In contrast, class 3 antibodies bind epitopes distinct from the ACE2-binding site but can potently neutralize. Class 4 antibodies are generally less potently neutralizing and recognize epitopes that are distinct from the ACE2-binding site shielded in the down conformation. Interestingly, some antibodies, namely, S309 and SW186, recognize epitopes that include the N343 glycan (29, 30), raising the possibility that this glycan might play a dual role in antibody recognition, either as a shield or as a component of an epitope.

The co-expression of SARS-CoV-2 S with envelope-deficient viruses such as HIV-1 produces pseudotyped viruses capable of infecting ACE2-expressing cells and is widely used as a surrogate to study viral entry and neutralization by antibodies (31, 32). To comprehensively understand the role of S glycans in viral infectivity and antigenicity, we individually mutated each of the 22 N-linked glycosylation sites in the S protein as well as two O-linked sites (T323 and S325) in the RBD. We found mutations introduced at many glycosylation sites in the NTD and RBD reduced pseudotype infectivity, and the magnitude of this effect was predicted by the magnitude of the loss of S incorporation into virions. Furthermore, while the S protein levels in virions had little effect on neutralization sensitivity, the presence or absence of a glycan on N343 in RBD affected sensitivity to several monoclonal antibodies cloned from convalescent individuals. Moreover, the glycan at N343 reduced neutralization sensitivity to polyclonal antibodies from convalescent individuals, but this evasive effect imparted by glycosylation was overcome by plasma antibodies from the same individuals who were subsequently vaccinated.

## RESULTS

### Levels of virion-incorporated SARS-CoV-2 S protein and pseudotyped HIV-1 particle infectiousness

**Fig 1:**
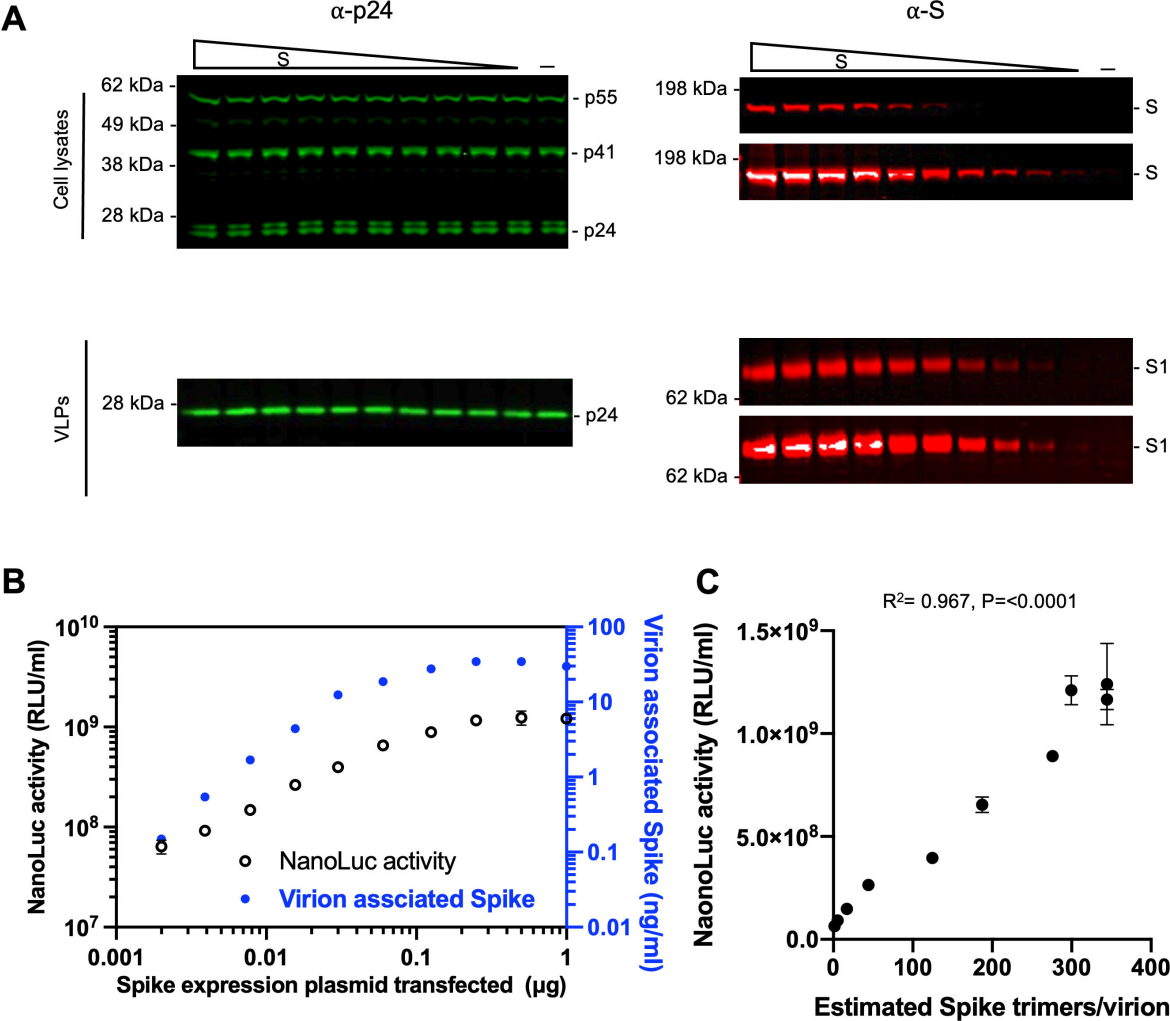
The effect of S protein level on pseudotype infectiousness. (**A**) Western blot analysis of 293T cell lysates (upper panel) or virions (lower panel) at 48 hours after transfection with various amounts (0, 2, 3.9, 7.8, 15.6, 31.2, 62.5, 125, 250, 500, or 1,000 ng) of a wild-type (WT) B.1 S expression plasmid along with envelope-deficient HIV-1 proviral plasmid expressing NanoLuc luciferase. Each S blot was scanned twice, at low intensity (*upper*) and high intensity (*low*er), respectively. Representative of three independent experiments. (**B**) Characterization of virions pseudotyped with S expression plasmids. Infectiousness (on the left *y*-axis) was quantified by measuring NanoLuc luciferase activity (RLUs) following infection of 293T cells expressing ACE2 (293T/ACE2.cl22) in 96-well plates with pseudotyped viruses. The S1 incorporated into viruses (on the right *y*-axis) was determined by quantitative western blot, using purified recombinant S-6P-NanoLuc proteins as standard, representative of three independent experiments. The mean and range of two technical replicates are shown. (**C**) Characterization of virions pseudotyped with S expression plasmids as in (**B**). Virus infectiousness (on the *y*-axis) was plotted against S trimers per virion (on the *x*-axis), which was determined by quantitative western blot, using purified recombinant S-6P-NanoLuc proteins as standard. Representative of three independent experiments. *R*^2^ and *P*-values were calculated using simple linear regression.

While SARS-CoV-2 S pseudotyped HIV-1 has been widely utilized to study S-mediated viral entry and neutralizing activities by antibodies, the extent to which the amount of S protein on virions affects particle infectivity and neutralization sensitivity is not fully understood. To address these questions, we co-transfected 293T cells with various amounts of a vector expressing S (pSARS-CoV-2_Δ19_) from the ancestral B.1 variant, which seeded the pandemic, along with an envelope-deficient HIV-1 proviral plasmid encoding NanoLuc. The number of SARS-CoV-2 spikes incorporated into HIV-1 pseudotype virions was estimated using purified recombinant S protein [S-6P-NanoLuc (33)] and recombinant p24 capsid (CA) proteins as standards on near-IR fluorescent western blotting (LiCor) and assuming 1,500 CA protein subunits per mature HIV-1 particle (34, 35) (Fig. S1A). While the amount of S expression plasmid used for transfection had little effect on the number of virions produced (Fig. 1A), the amount of S protein incorporated into virions correlated with the amount expressed in cells. S incorporation into virions reached a plateau (~30 ng S/mL in virions or around 300 S trimers per virion) when 0.125 or 0.25 µg of a B.1 S expression plasmid was co-transfected with the HIV-1 proviral plasmid (Fig. 1A through C).

To assess the effect of the number of spikes on pseudotype infectiousness, the titers of pseudotyped viruses were measured on 293T cells expressing ACE2 (293T/cl.22). Co-transfection with as little as 2 ng of S expression plasmid produced virions that induced 1,000× the level of luciferase observed with “bald virions,” indicating that small amounts of S protein (0.15 ng/mL or a mean of ~1–2 S trimers per virion) were sufficient to mediate viral entry (Fig. S2; Fig. 1B and C). Nevertheless, pseudotype virion infectivity increased with increasing spike numbers. Indeed, infectivity and the number of spikes were approximately linearly correlated, in the range 1–300 spikes per virion (Fig. 1C). Of note, these spike numbers are comparable to the average number of S trimers on authentic SARS-CoV-2 (strain Bavpat1/2020) virions (25–127 prefusion spikes per virion) (36) and higher than the numbers of gp120 trimers on HIV-1 virions (37).

### Reduced infectious virion yield conferred by SARS-CoV-2 S glycosylation site removal

**Fig 2:**
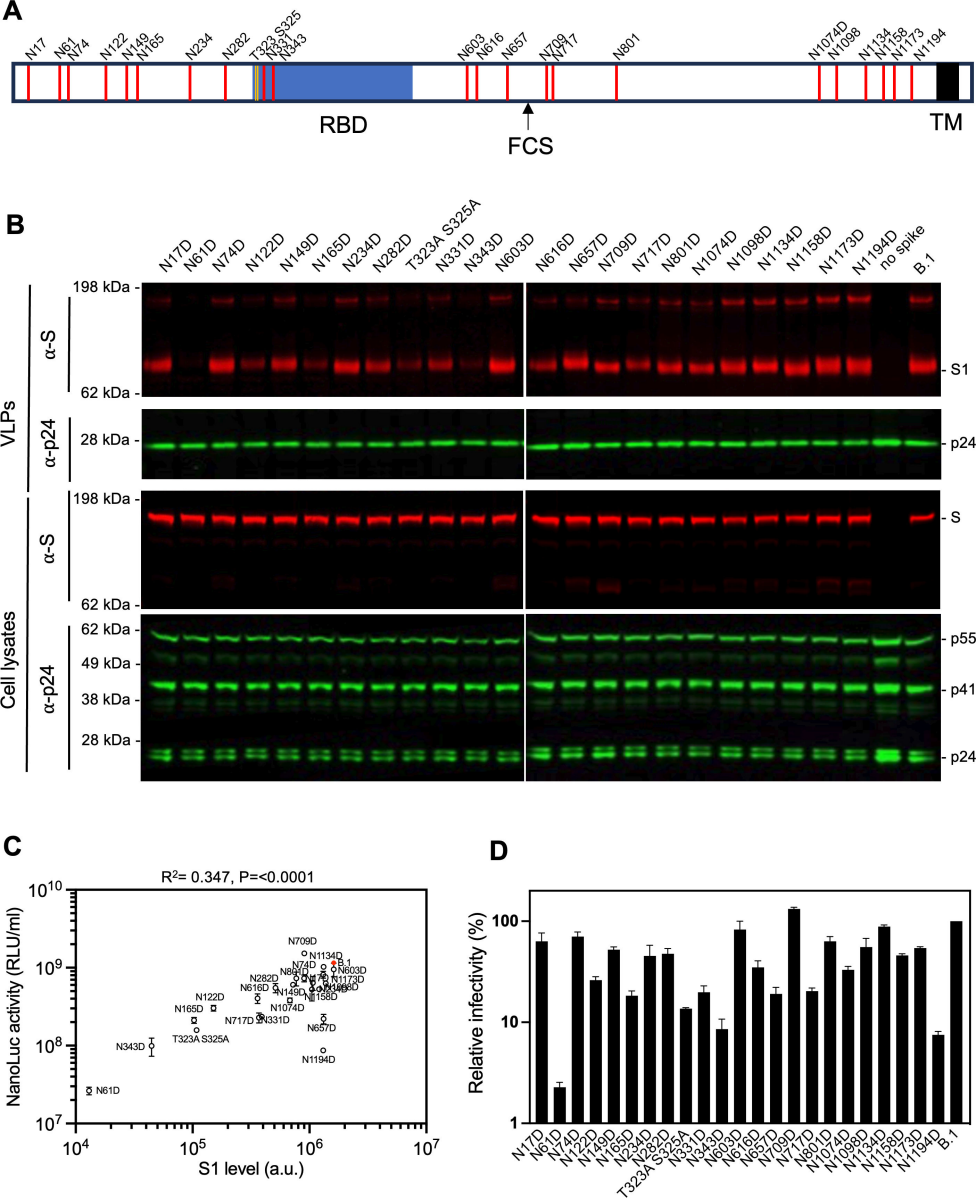
The impact of glycosylation site mutations on S incorporation and particle infectivity (**A**) Schematic representation of glycosylation sites on SARS-CoV-2 B.1 S with the RBD furin cleavage site and transmembrane domain indicated. N-linked sites are shown in red while O-linked sites within the receptor-binding domain are shown in orange. (**B**) Western blot analysis of 293T cell lysates (lower panels) or virions (upper panels) at 48 hours after transfection with 1 µg of glycosylation site mutants or wild-type B.1 S expression plasmid along with envelope-deficient HIV-1 proviral plasmid expressing NanoLuc. Representative of two independent experiments. (**C**) Characterization of virions pseudotyped with S expression plasmids. Infectiousness (on *y*-axis) was quantified by measuring NanoLuc luciferase activity (RLUs) following infection of 293T/ACE2.cl22 cells in 96-well plates with pseudotyped viruses. The S1 incorporated into viruses (on *x*-axis) was determined by quantitative western blot. Glycosylation site mutants are shown in black, and wild-type S is shown in red. The mean and range of two independent experiments are plotted. *R*^2^ and *P*-values calculated using simple linear regression. (**D**) Relative infectivity of glycosylation site mutants. Infectiousness was quantified as in (**C**) then normalized to WT (set at 100%). The mean and range of two independent experiments are plotted.

To investigate the contribution of glycosylation to S protein function, we generated 22 S substitution mutants, each containing a single Asn to Asp (N to D) substitution at one of the 22 N-glycosylation sequons on the B.1 S variant (Fig. 2A). Additionally, we generated a mutant with Ala replacements at potential O-linked sites T323 and S325 in the receptor-binding domain (13). None of these substitutions affected the levels of the S protein in transfected cells, but some of them reduced S incorporation into virions (Fig. 2B). Specifically, N61D, N122D, N165D, N343D, and T323A S325A substitutions that fall within S1, which includes the NTD and RBD, exhibited 10-fold or greater reductions in the levels of virion-associated S protein (Fig. 2B). Conversely, removal of the glycosylation sites in S2 had either no effect or minor effects on S protein incorporation into virions (Fig. 2A and B). Measurements of the yield of infectious pseudotyped particles carrying each of the substitutions indicated that several substitutions in the NTD and RBD markedly reduced infectivity. For example, the N61D substitution reduced infectivity by almost 50-fold (Fig. 2C and D; Fig. S2) while substitutions at glycosylation sites in the RBD, namely, N331D, N343D, and T323A S325A, resulted in 5- to 10-fold reduction in infectivity (Fig. 2C and D; Fig. S2). Across the glycosylation site mutant panel, the amount of S protein in virions was correlated with infectivity (Fig. 2C), suggesting a potential role for most glycans during synthesis or assembly of S trimers or their incorporation into virions, rather than in S protein function after virion incorporation. Nevertheless, two substitutions (N1194D and N657D) did not change the amount of S protein in virions but substantially reduced infectivity (Fig. 2B; Fig. S2). This finding suggests a functional role for N1194 and N657 glycans in S conformation or stability on virions or function during viral entry. Overall, we conclude that a subset of glycosylation sites is important for the incorporation of S protein (B.1) into virions, while others are required for optimal particle infectivity.

To attempt to understand how glycans might affect S protein incorporation into virions, we compared the subcellular distribution of wild-type S (B.1) with that of the two mutants with a pronounced deficit in virion incorporation, namely, N61D in the NTD and N343D in RBD (Fig. 2B). Immunofluorescence analysis of permeabilized cells suggested that the intracellular levels of the S proteins were not altered by glycan removal at either of these positions, in agreement with western blot analyses (Fig. 2A; Fig. S3). However, immunofluorescence analysis of non-permeabilized cells showed that the S protein levels on the surface of transfected cells were dramatically decreased for the N61D and N343D mutants (Fig. S3). To determine whether the S tail truncation (Δ19) used in the aforementioned experiments compromised S trafficking, the same WT and glycosylation site mutations were introduced into the full-length B.1 S protein and the same analyses performed. In agreement with the findings with the corresponding tail-truncated forms, the N61D and N343D substitutions in the full-length S protein had little effect on total protein expression levels but reduced cell surface levels (Fig. S3 and S4). Notably however, while the full-length S protein was clearly detected on the cell surface (Fig. S3), it was barely detected in virions (Fig. S4), consistent with previous findings that partial removal of the S cytoplasmic tail is necessary to generate highly infectious pseudotypes (32). Overall, we conclude that certain glycans are important for correct subcellular distribution of the SARS-CoV-2 S protein irrespective of whether the cytoplasmic tail is truncated to enable virion incorporation.

### Impact of spike density and RBD glycosylation on antibody neutralization sensitivity

**Fig 3:**
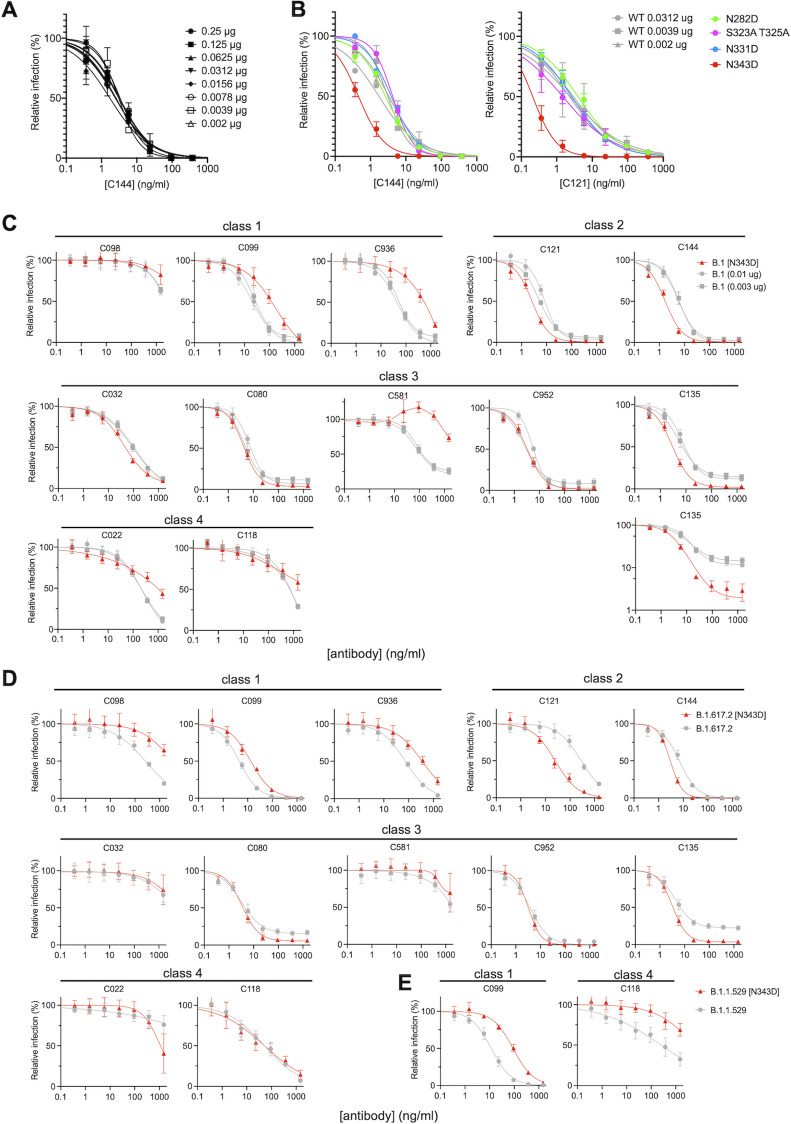
Neutralization sensitivity to monoclonal antibodies is affected by N343D S substitution. (**A**) Quantification of neutralization of pseudotyped virions from cells transfected with various amounts of wild-type B.1 S expression plasmid along with envelope-deficient HIV proviral plasmid expressing NanoLuc in the presence of the indicated concentrations of the class 2 neutralizing monoclonal antibody C144. Infectivity was quantified by measuring NanoLuc luciferase levels (RLU). The mean and range of two technical replicates are shown. (**B**) Quantification of glycosylation site mutant N282D, T323A S325A, N331D, and N343D or wild-type B.1 S pseudotyped virus infection in the presence of the indicated concentrations of the class 2 neutralizing monoclonal antibody C144 (*left*) or C121 (*right*). Infectivity was quantified by measuring NanoLuc luciferase levels (RLU). The mean and range of two technical replicates are shown. (**C**) Quantification of neutralization of glycosylation site mutant N343D in the background of furin uncleavable (R683G) B.1 S pseudotyped virus infection in the presence of the indicated concentrations of a panel of monoclonal antibodies, including class 1 (C098, C099, and C936), class 2 (C121 and C144), class 3 (C032, C080, C135, C581, and C952), and class 4 (C022 and C118) antibodies. As controls, glycosylation intact S expression plasmid (WT in the R683G background) was transfected at two doses, 10 or 3 ng, and the neutralization sensitivity of the resulting viruses was assessed in parallel. The mean and range of two independent experiments (two technical replicates in each experiment) are shown. The C135 neutralization graph is depicted with both a linear and a logarithmic *y*-axis to clearly show the effects of glycosylation on the completeness of neutralization at high antibody concentrations. (**D**) Quantification of neutralization of glycosylation site mutant N343D in the background of furin uncleavable (R683G) delta (B.1.617.2) S pseudotyped virus infection in the presence of the indicated concentrations of a panel of monoclonal antibodies, including class 1 (C098, C099, and C936), class 2 (C121 and C144), class 3 (C032, C080, C135, C581, and C952), and class 4 (C022 and C118) antibodies. The mean and range of three independent experiments (two technical replicates in each experiment) are shown. (**E**) Quantification of neutralization of glycosylation site mutant N343D in the background of furin uncleavable (R683G) omicron BA.1 (B.1.1.529) S pseudotyped virus infection in the presence of the indicated concentrations of a panel of monoclonal antibodies, including class 1 (C099) and class 4 (C118) antibodies. The mean and range of two independent experiments (two technical replicates in each experiment) are shown.

To determine the effect of glycan removal on sensitivity to neutralizing antibodies, we focused on glycosylation sites in the RBD (N331 and N343) and one site adjacent to the RBD (N282), as the RBD is the major target of neutralizing antibodies. Because these glycosylation sites affected the level of S incorporation into virions, we first tested the effect of SARS-CoV-2 spike density on neutralization sensitivity, as this parameter could be a potential confounder. We harvested pseudotyped virions from cells transfected with varying amounts (from 2 ng to 1 µg) of wild-type B.1 S expression plasmid and tested their sensitivity to C144, a potent class 2 neutralizing human monoclonal antibody cloned from a convalescent individual (25). Notably, varying the levels of WT S protein on virions over a wide range (Fig. 1B) had no discernable effect on neutralization sensitivity to C144 (Fig. 3A).

We next tested the neutralization sensitivity of the RBD glycosylation site mutants bearing Asn to Asp mutation at N-linked sites (N331D and N343D) or proximal (N282D) to the RBD or alanine substitutions at O-linked sites T323 and S325. To provide matched control viruses with approximately similar numbers of S trimers and similar levels of infectivity, neutralization of the glycosylation site mutants was compared to virions generated with an appropriate level of the WT B.1 S protein. We compared the neutralization sensitivity of WT and mutant B.1 virions to C144 and another potent class 2 neutralizing antibody, C121, both of which target the ACE2-binding site. While the N282D, T323/S325A, and N331D mutant pseudotypes exhibited neutralization sensitivities that were similar to the WT pseudotype, the N343D substitution conferred significantly increased neutralization sensitivity to both C144 and C121 (Fig. 3B). Specifically, the half-maximal inhibitory concentration (IC_50_) of C144 was reduced from 1.8 to 3.4 ng/mL (against the WT pseudotype) to 0.45 ng/mL (against the N343D pseudotype), while the C121 IC_50_ was reduced from 2.3 to 2.9 ng/mL (against the WT B.1 pseudotype) to 0.21 ng/mL (against the N343D pseudotype) (Fig. 3B).

### Positive and negative effects of RBD glycosylation on sensitivity to human monoclonal antibodies

To determine the effects of glycosylation more broadly on SARS-CoV-2 sensitivity to neutralizing antibodies, we used pseudotyped viruses bearing B.1 S proteins with R683G substitution, which ablates the furin cleavage site. This substitution does not affect S incorporation into virions (Fig. S1B) but enhances particle infectivity (7). The glycosylation site mutations had a smaller effect on S incorporation into virions in the R683G context (Fig. S5A). Nevertheless, transfection of cells with 1 µg of N282D, T323A S325A, and N331D B.1 S expression plasmids generated pseudotyped viruses with S protein amounts and infectious properties comparable to those generated by transfection of 30–100 ng of WT B.1 S expression plasmid (Fig. S5A and B). The transfection of cells with 1 µg of the N343D S expression plasmid yielded pseudotyped virus particles carrying a similar amount of S protein to those generated by cells transfected with 10 ng of the R683G B.1 S protein expression plasmid (Fig. S5A), while the particle infectivity was similar to those from cells transfected with 3 ng of the WT R683G expression plasmid (Fig. S5B). To evaluate the effect of glycosylation site mutations on sensitivity to neutralizing antibodies, we first generated B.1 WT and N343D mutant virion stocks bearing similar amounts of S protein and then assessed their susceptibility to neutralization by a panel of RBD-specific human monoclonal antibodies of the various neutralizing classes (24) recovered from convalescent individuals, including antibodies from class 1 [C098 (26), C099 (26), and C936 (27)], class 2 [C121 (25), C144 (25)], class 3 [C032 (25), C080 (26), C135 (25), C581 (27), and C952 (24)], and class 4 [C022 (25, 28), C118 (25, 28)].

Of class 1 antibodies, C098 had only weak neutralizing activity, whereas its clonal relative C099 was potent [IC_50_ = 21.3 ng/mL against B.1 WT (10 ng) or 24.5 ng/mL against B.1 WT (3 ng)], and its activity was resilient to many naturally occurring mutations in the RBD (7). The N343D mutation decreased the neutralization sensitivity to C099 by sevenfold, i.e., IC_50_ was increased to 176.9 ng/mL (Fig. 3C). Neutralization sensitivity to a third unrelated class 1 antibody, C936, was reduced by almost 10-fold, and IC_50_ was increased from 41.1 ng/mL against B.1 WT (10 ng) or 54.9 ng/mL against B.1 WT (3 ng) to 467.1 ng/mL against B.1 N343D (Fig. 3C).

In contrast to the class 1 antibodies, pseudotyped virus sensitivity to two class 2 antibodies was increased for the N343D mutant compared to WT. As was the case in the context of the furin-cleavable S protein (Fig. 3B), the N343D mutation in R683G S increased neutralization sensitivity to C121 and C144. The IC_50_ of C121 was reduced from 4.8 to 8.1 ng/mL (against the B.1 WT) to 2.6 ng/mL (against the B.1 N343D), while the C144 IC_50_ was reduced from 5.7 to 6.1 ng/mL (against the B.1 WT) to 1.7 ng/mL (against the B.1 N343D) (Fig. 3C).

Class 3 antibodies, which bind epitopes distinct from the ACE2-binding site, showed a complicated pattern of effects in response to the N343D substitution. Some class 3 antibodies, including C032, C080, and C952, inhibited the WT and N343D mutant pseudotypes with approximately the same potency. A different class 3 antibody C135, which exhibited incomplete neutralization of the WT pseudotypes at high concentrations despite exhibiting low IC_50_ (IC_50_ = 8.3–11.5 ng/mL), was able to achieve almost complete neutralization of the N343D pseudotypes at high concentrations (Fig. 3C). In contrast, another class 3 antibody C581 showed reduced potency against the B.1 N343D mutant, i.e., the IC_50_ was >2,000 ng/mL against N343D compared to 70.0–76.6 ng/mL against the wild-type B.1 S pseudotype (Fig. 3C).

For two class 4 antibodies, C022 and C118, the N343D substitution affected the slope of the neutralization curves and conferred partial resistance at high antibody concentrations (Fig. 3C). For example, C022 at 2,000 ng/mL almost completely neutralized the wild-type B.1 S pseudotypes but only inhibited the B.1 N343D pseudotypes by ~50%. A similar trend was seen for a second class 4 antibody, C118.

Given the effects of the N343D substitution on B.1 sensitivity to neutralizing antibodies, we also determined the effect of this substitution in the context of S variants delta (B.1.617.2) and omicron BA.1 (B.1.1.529). The introduction of the N343D mutation into the delta (B.1.617.2) S resulted in sixfold reduction in pseudotype infectivity (Fig. S6A) and affected neutralization sensitivity to the four classes of antibodies in a manner that was similar to the effects on the B.1 pseudotype (Fig. 3D). Specifically, the N343D substitution decreased sensitivity to class 1 antibodies C098, C099, and C936, with increases in IC_50_ values of 12-fold, 3-fold, and 5-fold, respectively (Fig. 3D). The N343D substitution also rendered the delta (B.1.617.2) more sensitive to class 2 antibodies C121 and C144, with IC_50_ values decreasing by 11-fold and 2-fold, respectively (Fig. 3D). Effects on neutralization sensitivity for class 3 and 4 antibodies were small (Fig. 3D), but as was the case for the B.1 pseudotype, incomplete neutralization by the C135 antibody (and to some extent the C080 antibody) was rendered more complete by the N343D substitution. Introduction of the N343D substitution into the omicron (B.1.1.529) S resulted in an even more pronounced decrease in pseudotype infectivity of around 50-fold (Fig. S6B). Most of the antibodies lacked neutralizing activity against the omicron BA.1 pseudotype, and introduction of the N343D substitution did not reverse the intrinsic resistance of omicron BA.1 to those antibodies (Fig. S7). For the two antibodies that neutralized omicron BA.1, the N343D substitution reduced neutralization sensitivity by 8-fold (for C099) and 22-fold (for C118, Fig. 3E).

Overall, the N343D substitution had a range of positive and negative effects on neutralization sensitivity that varied greatly dependent on the nature of the particular monoclonal antibody tested. In general, the presence of the glycan increased sensitivity to class 1 antibodies but decreased sensitivity to class 2 antibodies.

**Fig 4:**
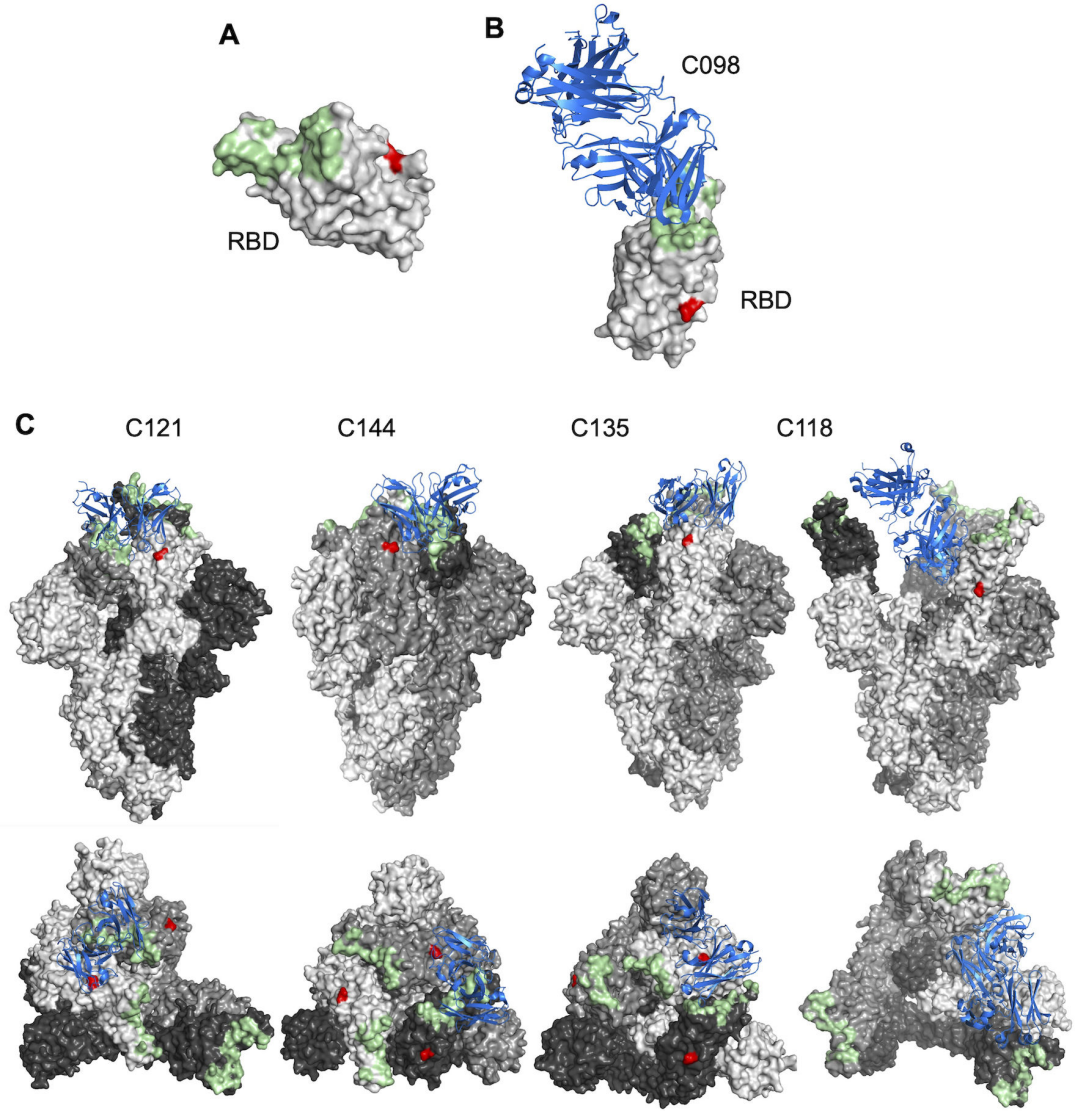
Proximity of SARS-CoV-2 S N343 to antibody binding sites. (**A**) Surface representation of the RBD in an X-ray crystal structure (PDB ID: 7K8M). ACE2-binding site and the N343 glycosylation site are highlighted in pale green and red, respectively. (**B**) View of C098 Fab variable domains (blue) binding to spike (gray). ACE2-binding site and N343 are highlighted in pale green and red, respectively. The structure illustrated herein is C098 (PDB ID: 7N3I). (**C**) Views of antibody Fab variable domains (blue) binding to S trimers (each trimer subunit is colored in a different shade of gray). ACE2-binding site and N343 are highlighted in pale green and red, respectively. The antibody structures illustrated include C121 (PDB ID: 7K8X), C144 (PDB ID: 7K90), C135 (PDB ID: 7K8Z), and C118 (PDB ID: 7RKV). Side (upper) and top (lower views for each structure are shown).

To better understand the molecular basis for the impact of N343 glycan on antibody neutralization, we inspected the structures of some of the aforementioned antibodies in complex with ancestral S (B.1) (Fig. 4A through C). The glycosylation site at N343 (shown in red in Fig. 4A through C) is distinct from the binding site of the class 1 antibody C098—suggesting that the effects of the glycan on sensitivity to class 1 antibodies are mediated through effects of the glycan on RBD conformational dynamics and epitope exposure (Fig. 4B). In contrast, for the class 2 antibodies C121 and C144, N343 is proximal to the antibody bound to the neighboring S subunit (Fig. 4C). Since the removal of the glycan increased sensitivity to these antibodies (Fig. 3C), it is likely that the N343 glycan partly shields these class 2 antibody epitopes. For C135, a class 3 antibody, N343 protrudes toward the antibody binding site on the same S subunit (Fig. 4), potentially explaining incomplete neutralization by this antibody (Fig. 3C). For the class 4 antibody C118, N343 is distal to the antibody binding site on S (Fig. 4C), suggesting that N343 glycan is not involved in direct antibody binding and likely exerts changes in neutralization through effects on RBD conformational dynamics.

Other RBD substitutions (T323A/S325A and N331D) did not alter the neutralization sensitivity to most monoclonal antibodies tested herein (Fig. S8 and S9). However, the N282D substitution, which lies outside the RBD, had a marginal effect on sensitivity to the class I antibodies C099 and C936 and the class 3 antibody C581 (Fig. S10).

### Effect of the N343 glycan on neutralization by polyclonal SARS-CoV-2 neutralizing plasma

**Fig 5:**
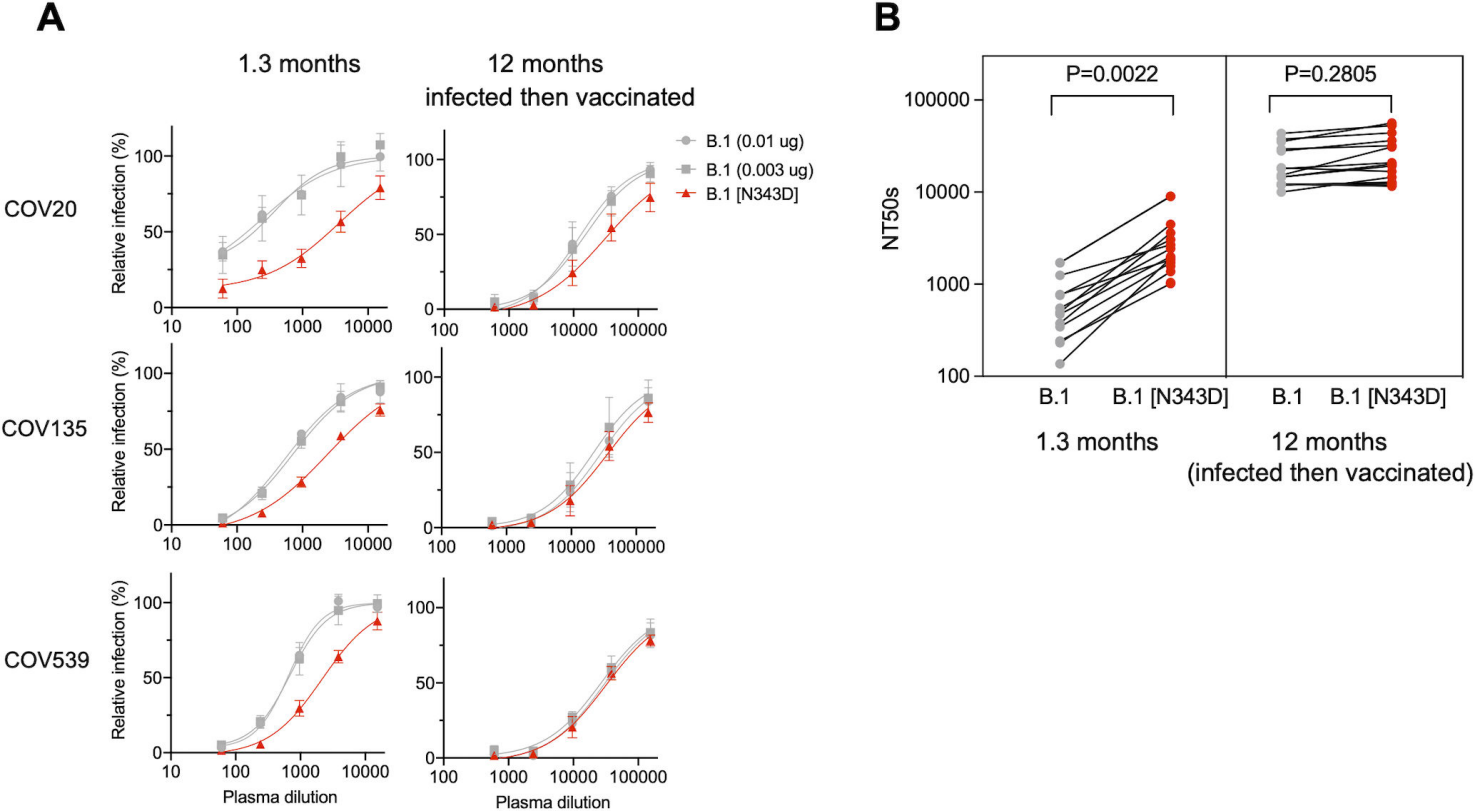
Neutralization sensitivity of N343D mutant to convalescent plasma. (**A**) Plasma neutralization of N343D or glycosylation site intact B.1 S [in the furin uncleavable (R683G) background] pseudotyped virus using 293T/ACE2.cl22 target cells. The convalescent plasma samples were collected at 1.3 and 12 months (infected then vaccinated) and diluted fourfold serially followed by incubation with viruses. As controls, glycosylation intact S expression plasmid (WT in the R683G background) was transfected at two doses, 10 or 3 ng, and the neutralization sensitivity of the resulting viruses was assessed in parallel. The mean and range of two technical replicates are shown. (**B**) Comparison of NT_50_ values for each of the 15 convalescent plasma samples collected at 1.3 and 12 months (infected then vaccinated) for the N343D or glycosylation intact B.1 S pseudotypes. *P*-values calculated using Welch’s corrected *t*-test.

Given that the N343D substitution exhibited different effects on sensitivity to neutralizing monoclonal antibodies, we next asked whether this substitution affected neutralization by polyclonal antibodies present in convalescent plasma. As with the monoclonal antibodies, we compared the neutralization sensitivity of N343D pseudotyped particles with that of WT pseudotyped particles containing the same amount of WT S protein or showing the same infectivity as the N343D pseudotype to convalescent plasma. We randomly selected 15 individuals from a cohort who were infected early in the pandemic with B.1-like SARS-CoV-2 strains and subsequently immunized with B.1-like S mRNA vaccines approximately 10 months after infection (27). Plasma samples from these individuals were collected at two time points: at 1.3 months after infection (prior to vaccination) and 12 months after infection (post-vaccination). The N343D mutant pseudotypes were more sensitive than the WT pseudotypes to convalescent plasma collected at 1.3 months after infection (Fig. 5A; Fig. S11; Table S1). Indeed, the 50% neutralization titers (NT_50_) were a mean of 5.1-fold greater for the N343D mutant as compared to WT pseudotypes (*P* = 0.0022) (Fig. 5B; Table S1). Notably, the difference in neutralization sensitivity between N343D mutant (mean NT_50_ = 26,179) and WT S pseudotypes (mean NT_50_ = 20,853) was negligible (*P* = 0.2805) when plasmas collected from the same individuals 1 year later and after subsequent vaccination were tested. Of note, these subsequently collected plasmas exhibited higher neutralization potency than those collected shortly after infection (Fig. 5A and B). We conclude that the glycan at N343 confers protection against neutralization by antibodies generated shortly after SARS-CoV-2 infection but that this effect is lost against antibodies from the same individuals who are subsequently vaccinated.

## DISCUSSION

While SARS-CoV-2 S pseudotyped viruses have been widely used as a surrogate to study S-mediated viral entry (32), little is known about the varying effect of S level on virions on particle infectivity and neutralization sensitivity. Previously, it was reported that approximately eight HIV-1 trimer–receptor interactions are required for HIV-1 to infect a target cell (38), a result that is broadly consistent with our finding that a small number (minimally an average of one to two spikes per virion) are sufficient for the detection of SARS-CoV-2 pseudotype infection. To achieve neutralization, monoclonal antibodies must encounter prefusion spikes and reduce the number of functional spike trimers below the threshold required for infection. We found that an increased level of S on pseudotyped virions is associated with increased infectivity but had little effect on neutralization sensitivity to monoclonal antibodies. This result suggests that monoclonal antibodies are in excess over functional spikes in a typical neutralization assay, and their measured potency is limited by their affinity, rather than by the amount of functional S protein in a pseudotype neutralization assay.

In this study, we found that glycosylation at several sites in the S NTD and RBD, including N61, N122, N165, N331, N343, T323, and S325, are required for full pseudotyped virus infectivity. Substitution of these glycosylation sites resulted in a reduction in pseudotype infectivity of about 10-fold or more and correlated with a reduction in S incorporation into virions. While it is unclear precisely how glycosylation on these sites drives S incorporation into virions, glycans could in principle affect protein stability and trafficking through the secretory pathway (39). We note, however, that the glycosylation site mutations did not affect steady-state levels of S in transfected cells. Compared with other glycosylation sites in the S1 domain, these sites are highly conserved among sarbecovirus S proteins (40). Likewise, the conservation of these glycosylation sites is observed among the major SARS-CoV-2 variant lineages, including alpha, beta, gamma, delta, and omicron (41). Indeed, N343D substitution in the delta and omicron BA.1 variant S impaired pseudovirus infectivity. Some studies, largely employing molecular dynamics simulations, have suggested that glycans on S proteins affect the conformational dynamics of the spike’s RBD “up” and “down” states (10, 42). In particular, the glycan at N343 stabilizes RBD states in a process termed “glycan gating” (22, 43, 44). Our findings show that the N-glycan at N343 affected incorporation into virions, suggesting that the conformational state might affect trimer assembly or stability, transit through the secretory pathway, or incorporation into pseudotyped virions. Indeed, our data show that cell surface levels of the N61D and N343D mutant S proteins were diminished. We note that differences in S trafficking could affect virion incorporation in lentiviral particles differently to authentic coronaviruses. Nevertheless, several studies have demonstrated that S-pseudotyped retroviruses accurately reflect the neutralization properties of authentic viruses (32, 33, 45).

The N343 glycan, either in the context of the ancestral strain B.1 or variants delta and omicron BA.1, also affected neutralization by RBD-targeted monoclonal antibodies, and the effect was largely dependent on the class to which antibodies belong. The N343 glycan is positioned on the RBD distal to the ACE2 and class 1 antibody (C098) binding site but increased sensitivity to class 1 antibodies (C098, C099, and C936), which bind only to “up” RBDs (24), suggesting that this effect is mediated through the impact of the glycan on RBD conformation. Conversely, the N343 glycan reduced sensitivity to class 2 antibodies, including C121 and C144, both of which can bind both “up” and “down” RBDs. Cryo-EM structures (24) (Fig. 4) show that N343 is located proximally to the C121 and C144 antibody bound to the neighboring subunit, in a manner that might interfere with antibody binding. Overall, our results are consistent with a model in which N343 glycan affects sensitivity to class 1 and class 2 antibodies by affecting the RBD conformational dynamics (43) and also potentially by sterically hindering class 2 antibody binding into neighboring RBDs in the “down” conformation (Fig. 4).

The N343 glycan is proximal to the binding sites of class 3 antibodies, and the N343D substitution had a range of effects on sensitivity to class 3 antibodies. Three clonally related antibodies, C032, C080, and C952, were unaffected by the N343D substitution, while substantial but opposing effects were seen for two others, C135 and C581. In the case of C135, the removal of the glycan reduced the fraction of virions that resisted neutralization at high antibody concentrations. A possible explanation for this phenomenon is that the N343 site is heterogeneously glycosylated, and some subfraction of the glycans occludes the C135 binding site. For C581, the removal of the N343 glycan had the opposite effect, reducing neutralization sensitivity. In this case, it seems likely that C581 mimics the properties of two previously described cross-reactive class 3 antibodies, S309 and SW186, for which the N343 glycan constitutes part of the antibody binding site (29, 30). Finally, for the two class 4 antibodies, the glycan at N343 affected the character of the neutralization curve. Since in these cases the antibody binding site is on the opposite face of the RBD to that of the glycan, it is likely that these effects are mediated through the alteration of spike conformational dynamics and exposure of the class 4 epitope, which is shielded in the RBD “down” conformation.

The N343 glycan reduced neutralization by convalescent plasma collected at 1.3 months after infection, echoing the effects on sensitivity to the C121, C144 (class 2), and C135 (class 3) antibodies. Conversely, neutralization by convalescent plasma from the same individuals after vaccination, collected at 12 months post-infection, was unaffected by the N343 glycan. The fact that neutralizing antibodies are relatively sensitive to glycan-mediated protection early after infection may be a reflection of the fact that the initial neutralizing response is based to a large extent on class 2 antibodies that are easily escaped (25, 33). Subsequent increases in antibody affinity and neutralizing potency and a shift in the RBD epitopes that are recognized, following months of affinity maturation and boosting by vaccination (7, 26, 27, 46), result in neutralizing antibodies that are mostly unaffected by the N343 glycan. In sum, while the SARS-CoV-2 N343 glycan affects both spike conformation and neutralization sensitivity shortly after infection, antibody evolution confers sufficient potency and breadth to combat glycan-mediated immune evasion.

## MATERIALS AND METHODS

### Antibodies and recombinant HIV p24

Antibodies used here are anti-SARS CoV-2 S antibody [1A9] (GeneTex, GTX632604) and anti-HIV capsid p24 (183-H12-5C, NIH AIDS Research and Reference Reagent Program). Secondary antibodies included goat anti-mouse conjugated to IRDye 800CW or IRDye 680 for western blot analysis. The recombinant HIV p24 protein was purchased from Abcam (ab43037).

### Plasmid construction

The plasmid expressing C-terminally truncated, human-codon-optimized SARS-CoV-2 B.1 S protein (pSARS-CoV-2_Δ19_) has been previously described (32). Using this plasmid as the template, asparagine at N-linked glycosylation sites was replaced by aspartate by overlap-extension PCR amplification with primers that incorporated the corresponding nucleotide substitutions. O-linked glycosylation sites in the RBD region (T323, S325) were replaced by alanine using the same strategy. The purified PCR products were then inserted into the pCR3.1 expression vector with NEBuilder HiFi DNA Assembly. Some mutations within or adjacent to the RBD region, including N282D, N331D, N343D, and T323A S325A, were also introduced in S bearing the R683G substation, which impairs the furin cleavage site and enhances particle infectivity. The delta (B.1.617.2) N343D and omicron (B.1.1.529) mutants were constructed using the same strategy, with the templates being the unmodified delta and omicron BA.1 S sequences, respectively.

### Cell lines

Human embryonic kidney HEK-293T cells (ATCC CRL-3216) and the derivative expressing ACE2, i.e., 293T/ACE2.cl22, were maintained in Dulbecco’s modified Eagle medium supplemented with 10% fetal bovine serum (Sigma F8067) and gentamycin (Gibco). All cell lines used in this study were monitored periodically to ensure the absence of retroviral contamination and mycoplasma.

### Generation of clarified SARS-CoV-2 pseudotyped virions and infectivity measurement

To generate HIV-1/NanoLuc-SARS-CoV-2 B.1 pseudotyped virions, 2 million 293T cells in 10 cm dish were transfected with 7.5 µg of HIV-1 proviral plasmids expressing NanoLuc along with increasing amounts (0, 2, 3.9, 7.8, 15.6, 31.2, 62.5, 125, 250, 500, or 1,000 ng) of WT B.1 or 1,000 ng of glycosylation site mutant SARS-CoV-2 B.1 expression plasmids (pSARS-CoV-2_Δ19_). In the transfection experiment to generate viruses bearing R683G substitution, 1, 3, 10, 30, 100, and 300 ng of expression plasmid were used instead. The total amount of DNA was held constant by supplementing the transfection with an empty expression vector. To generate HIV-1/NanoLuc-SARS-CoV-2 delta (B.1.617.2) and omicron (B.1.1.529) pseudotyped virions, 2 million 293T cells in 10 cm dish were transfected with 7.5 µg of HIV-1 proviral plasmids expressing NanoLuc along with 1 µg of WT (B.1.617.2 or B.1.1.529) or N343D mutant SARS-CoV-2 expression plasmids [B.1.617.2 (N343D) or B.1.1529 (N343D)] bearing R683G. Cells were harvested at 48 hours post-transfection and subjected to western blot analysis. Virus-containing supernatant was filtered (0.2 µm), and, to remove cell debris, clarified by Lenti-X (TaKaRa). Particle infectivity was measured as previously described (32). Briefly, viral stocks were threefold serially diluted and added to 293T/ACE2 cl.22 in 96-well plates. Cells were then harvested at 48 hours post-infection for measuring NanoLuc activity (Promega). The number of S trimers per virion was estimated using the following formula: S ng/mL/78.3 × 1,500/(p24 ng/mL/24)/3.

### Western blot analysis

Cell lysates were separated on NuPage Novex 4%–12% Bis-Tris Mini Gels (Invitrogen). Proteins were blotted onto nitrocellulose membranes. Thereafter, the blots were probed with primary antibodies and followed by secondary antibodies conjugated to IRDye 800CW or IRDye 680. Fluorescent signals were detected and quantitated using an Odyssey scanner (LI-COR Biosciences).

### Immunofluorescence assay

To determine whether glycosylation site mutations affect S protein trafficking, HT1080 cells were transfected with vectors expressing WT S (B.1) or N61D or N343D either in the full-length background or tail-truncated form (Δ19). Two days later, cells were fixed with 4% paraformaldehyde/PBS and treated with 10 mM glycine. To visualize, intracellular S protein cells were permeabilized using 0.2% Tween 20. S protein was detected with mouse anti-S antibody (GeneTex, Cat. No. GTX632604, clone 1A9, 1/100 dilution) followed by staining with goat anti-mouse Alexa Fluor 488 (Thermo Fisher Scientific, cat A-11029, 1/500 dilution). Images were captured and deconvolved using a DeltaVision OMX SR imaging system (GE Healthcare).

### S-6P-NanoLuc protein purification

To express S-6P-NanoLuc proteins, Expi293 cells were transfected with S-6P-NanoLuc expression plasmids using ExpiFectamine 293 (Thermo Fisher Scientific). Four days later, the supernatant was harvested and loaded on Ni-NTA agarose, and after a thorough wash, S-6P-NanoLuc proteins were released after HRV 3C protease treatment.

### Neutralization assays

To measure the neutralizing activity of monoclonal antibodies, serial dilutions of antibodies beginning at 3 µg/mL were fourfold serially diluted in 96-well plates over seven dilutions. To determine the neutralizing activity in convalescent plasma, the initial dilution started at 1:30 (for plasma at 1.3 months) or 1:150 (for plasma at 12 months). Thereafter, SARS-CoV-2 S pseudotyped viruses were incubated with monoclonal antibodies or the convalescent plasma for 1 hour at 37°C in 96-well plates. The mixture was then added to 293T/ACE2cl.22 target cells seeded 1 day prior to infection, so the final starting dilutions were 1.5 µg/mL for monoclonal antibodies and 1:60 or 1:300 for plasma. Cells were then harvested 48 hours post-infection for NanoLuc luciferase assays.

### Human plasma samples and monoclonal antibodies

Monoclonal antibodies C022, C032, C080, C098, C099, C118, C121, C135, C144, C581, C936, and C952 used in this study were previously reported (24–28). The human convalescent plasma samples were obtained under protocols approved by Institutional Review Boards at Rockefeller University.

## SUPPLEMENTAL MATERIAL

Supplemental material mbio.01672-23-s0001.pdf Figures S1-S11 and Table S1.

ASM does not own the copyrights to Supplemental Material that may be linked to, or accessed through, an article. The authors have granted ASM a non-exclusive, world-wide license to publish the Supplemental Material files. Please contact the corresponding author directly for reuse.
