# Supplementary material for: SARS-CoV-2 spike glycosylation affects function and neutralization sensitivity
Source: mBio. 2024 Jan 9;15(2):e01672-23. doi: 10.1128/mbio.01672-23 (PMC10865855; doi:10.1128/mbio.01672-23)
Supplement: Supplemental material — Figures S1-S11 and Table S1. [file mbio.01672-23-s0001.pdf]

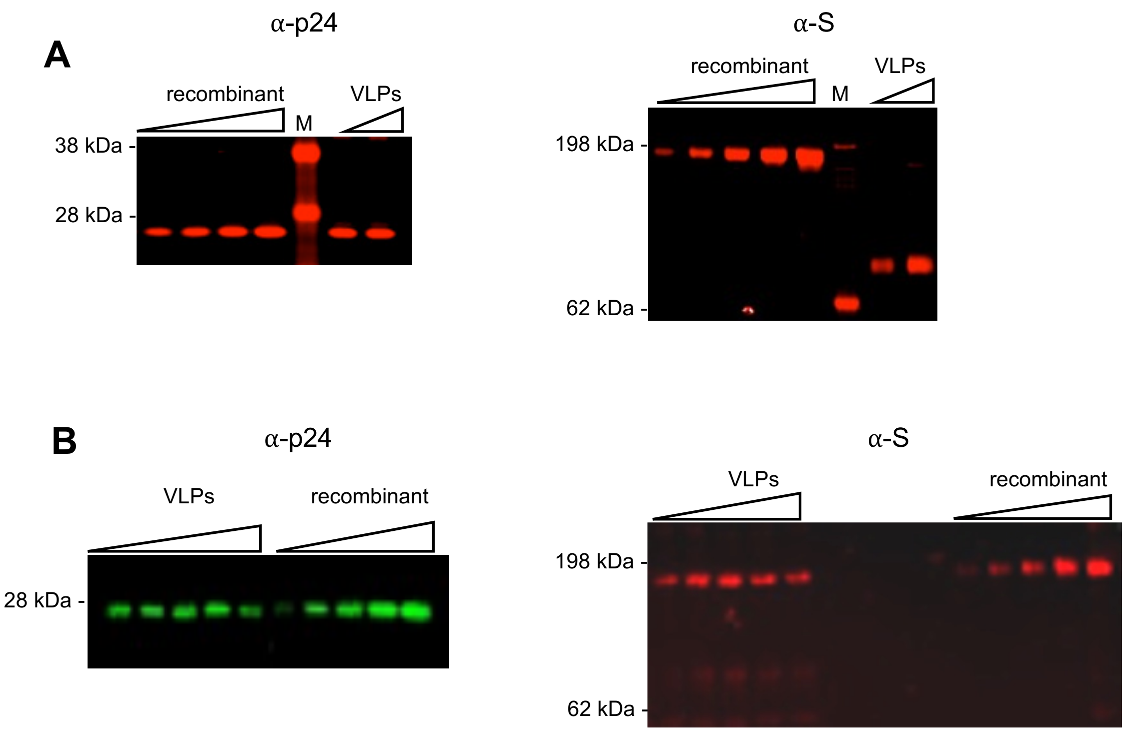

FIG S1 Western blot analysis of virions, using recombinant proteins as standard
(A) Western blot analysis of virions pelleted through 20% sucrose from 100 µl supernatant harvested at 48 hours after transfection with 0.0625 µg or 0.5 µg of wild-type S expression plasmid along with envelope-deficient HIV-1 proviral plasmid expressing NanoLuc luciferase. The blot was probed with an anti-p24 antibody and recombinant HIV p24 protein was used as a standard (1.0 ng, 2.0 ng, 4.0 ng, or 8.0 ng per lane) on the left, or with anti-S antibody using recombinant S-6P-nanoLuc as a standard (0.25 ng, 0.5 ng, 1.0 ng, 2.0 ng, or 4.0 ng per lane) on the right. Representative of two independent experiments.
(B) Western blot analysis of virions pelleted through 20% sucrose from 100 µl supernatant harvested at 48 hours after transfection with 0.008 µg, 0.024 µg, 0.073 µg, 0.22 µg, or 0.67 µg of wild-type S expression (furin uncleavable R683G background) along with envelope-deficient HIV-1 proviral plasmid expressing NanoLuc luciferase. The blot was probed with anti-p24 antibody using recombinant HIV p24 protein as standard (0.5 ng, 1.0 ng, 2.0 ng, 4.0 ng, or 8.0 ng per lane) on the left, or with anti-S antibody using recombinant S-6P-nanoLuc as standard (0.125 ng, 0.25 ng, 0.5 ng, 1.0 ng, or 2.0 ng per lane) on the right. Representative of two independent experiments.

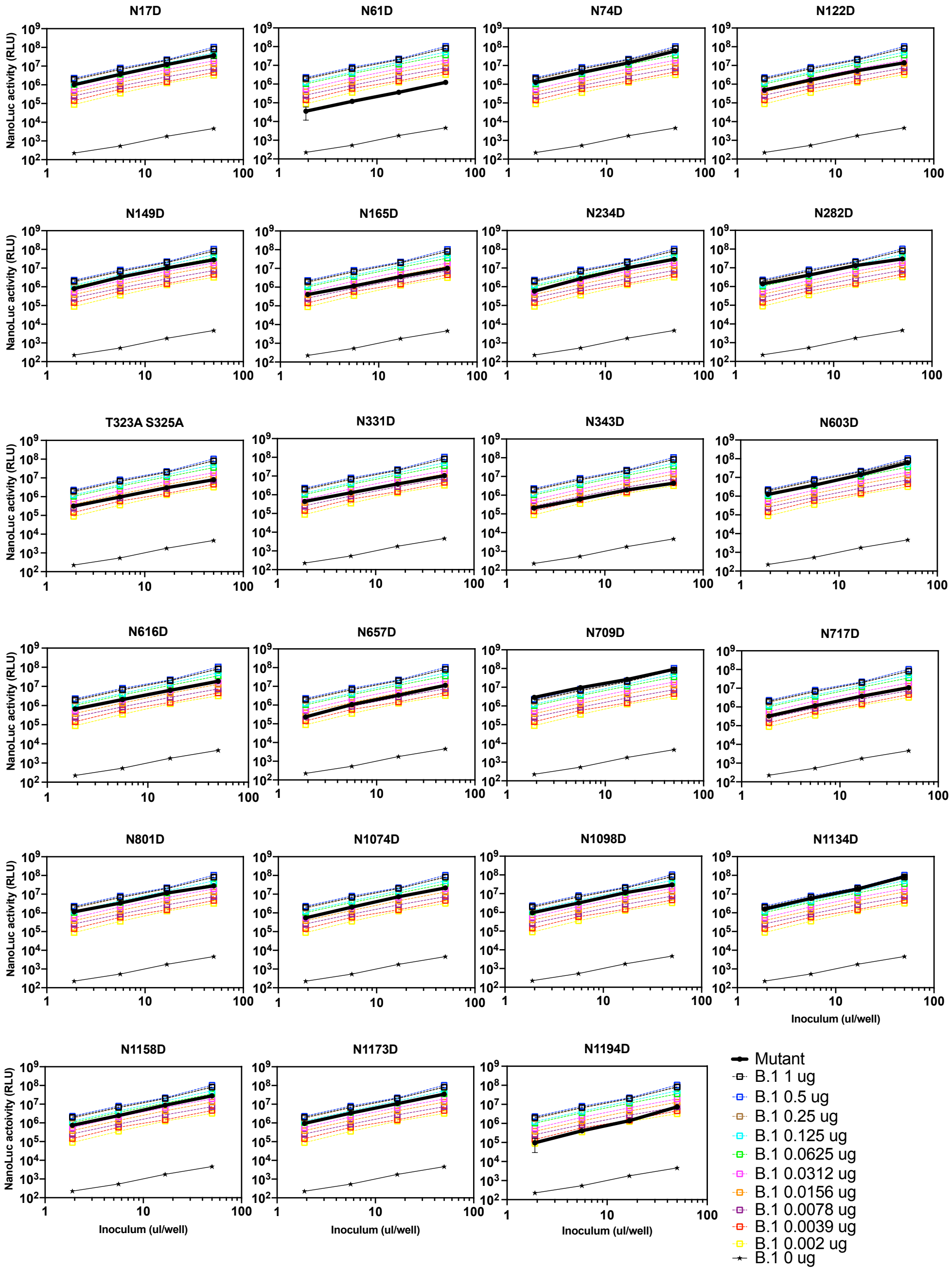

FIG S2 The impact of glycosylation site mutations on particle infectivity
Infectious virion measurements of for pseudotypes bearing glycosylation site mutant S proteins (bold black lines, 1 μg transfected S expression plasmid), compared with WT S pseudoytpes (dashed lines) collected from 293T cells transfected with various amounts of S expression plasmids. Infectivity was quantified by measuring NanoLuc luciferase activity (RLU). Virus generated in the absence of S (0 μg), shown in thin black line, was used as a background control. 293T/ACE2.cl22, as target cells, were infected with the indicated volumes of pseudotyped viruses in 96-well plates and harvested 48 hours post infection for NanoLuc luciferase assay. The mean and range deviation from two technical replicates are shown.

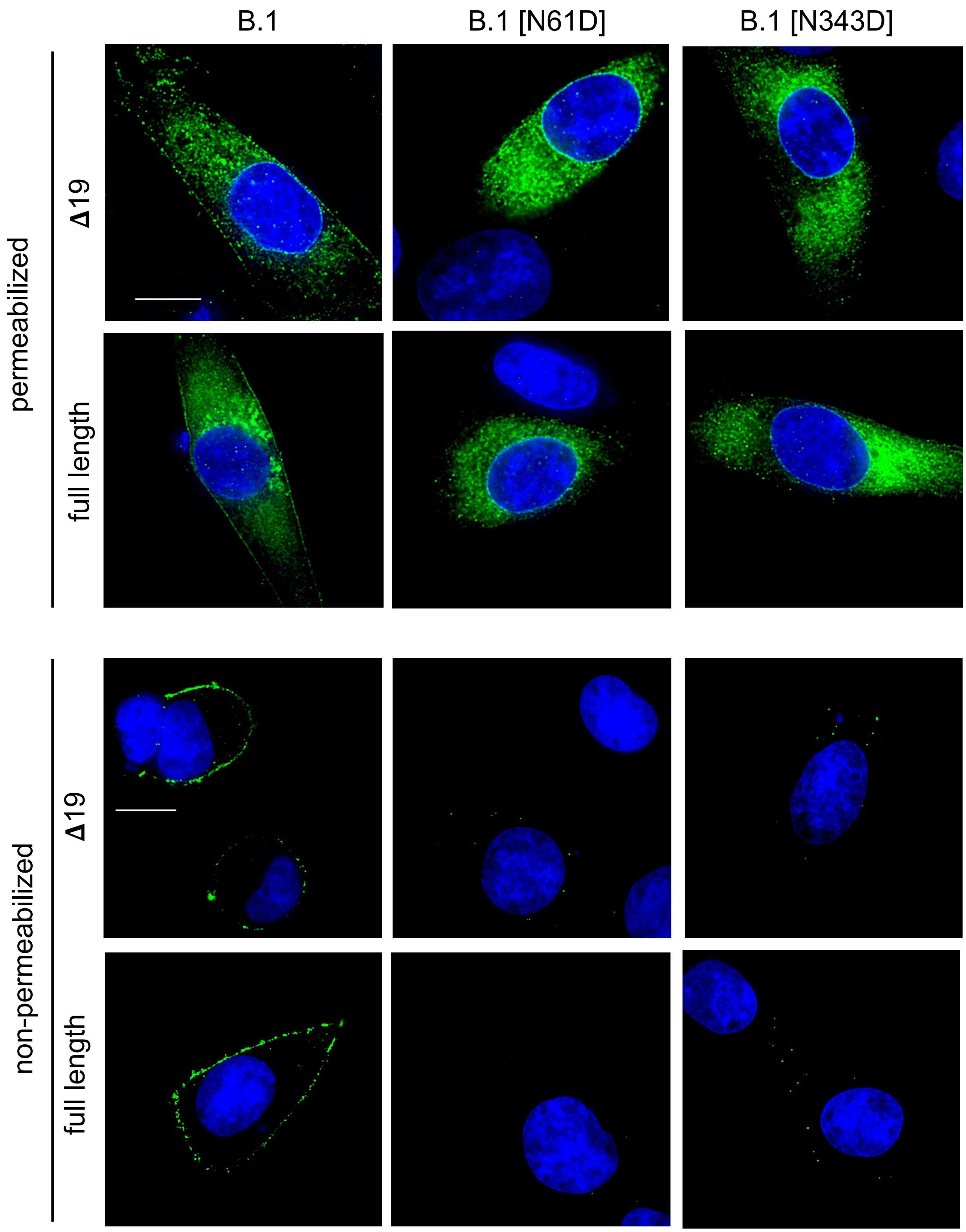

FIG S3 The impact of glycosylation site mutations on SARS-CoV-2 S trafficking
Localization of S (green) in HT1080 cells transfected with vectors expressing glycosylation site mutants N61D, N343D, or wild-type B.1 S, either tail-truncated or full length, respectively. Blue stain (DAPI) indicates cell nuclei. Scale bars, 10 µm. Each image is representative of 10 cells, imaged in two independent experiments.

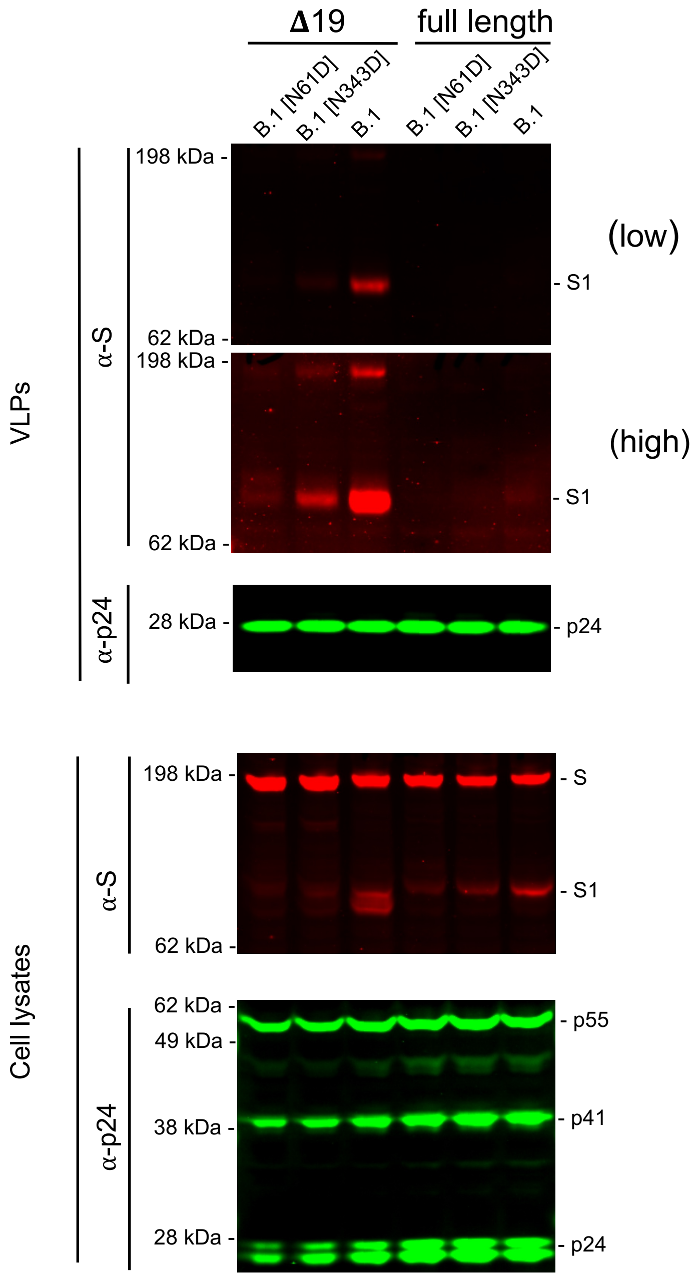

FIG S4 The impact of glycosylation site mutations on incorporation of the full-length SARS-CoV-2 S protein

Western blot analysis of 293T cell lysates (*lower* panels) or virions (*upper* panels) at 48 hours after transfection with vectors expressing glycosylation site mutants N61D, N343D, or wild-type B.1 S, either tail-truncated (*left*) or full-length (*right*), respectively, along with envelope-deficient HIV-1 proviral plasmid expressing NanoLuc. The blot showing S protein in virions is scanned at low intensity (low) and high intensity (high). Representative of two independent experiments.

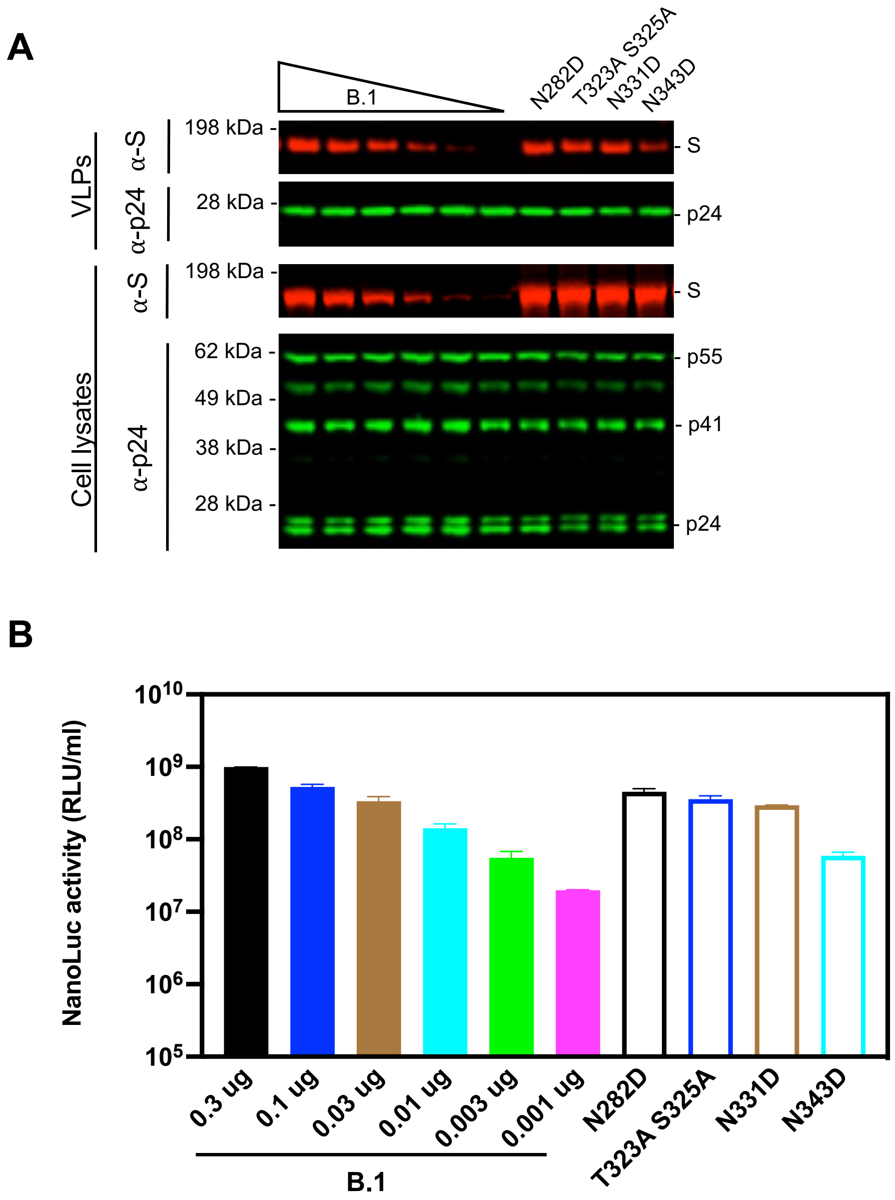

FIG S5 The impact of glycosylation site mutations in the furin uncleavable (R683G) background on B.1 S incorporation and particle infectivity

(A) Western blot analysis of 293T cell lysates (lower panel) or virions (upper panel) at 48 hours after transfection with various amounts of glycosylation site intact S expression plasmid (R683G background), or 1 μg of glycosylation site mutants (N282D, T323A S325A, N331D, or N343D) along with envelope-deficient HIV-1 proviral plasmid expressing NanoLuc.

(B) Infectivity was quantified by measuring NanoLuc luciferase activity (RLUs) following infection of 293T expressing ACE2 (293T/ACE2.cl22) in 96-well plates with pseudotyped viruses as depicted in (A). The mean and range of two technical replicates are plotted.

A

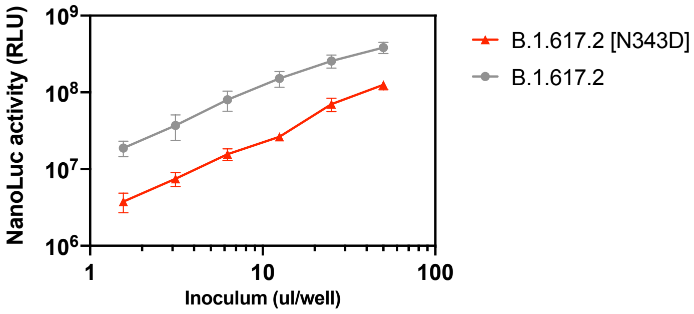

B

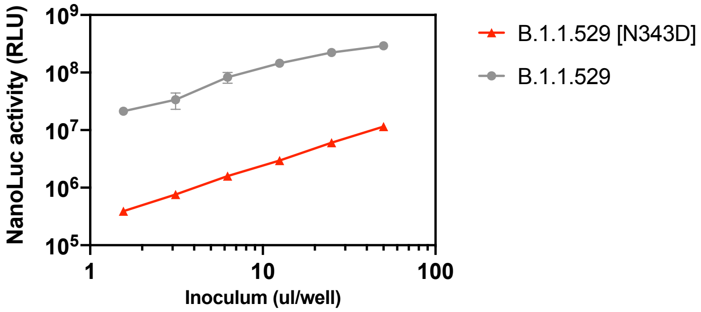

FIG S6 The impact of N343D substitution on virus infectivity pseudotyped with delta (B.1.617.2) or omicron (B.1.1.529)

(A) Infectious virion measurements of pseudotypes bearing delta (B.1.617.2) N343D S proteins (red line), compared with WT delta (B.1.617.2) S pseudoytpes (gray line) collected from 293T cells after transfection. Infectivity was quantified by measuring NanoLuc luciferase activity (RLU). The mean and range deviation from two independent experiments, two technical replicates in each experiment, are shown.

(B) Infectious virion measurements of pseudotypes bearing omicron (B.1.1.529) N343D S proteins (red line), compared with WT omicron (B.1.1.529) S pseudoytpes (gray line) collected from 293T cells after transfection. Infectivity was quantified by measuring NanoLuc luciferase activity (RLU). The mean and range deviation from two independent experiments, two technical replicates in each experiment, are shown.

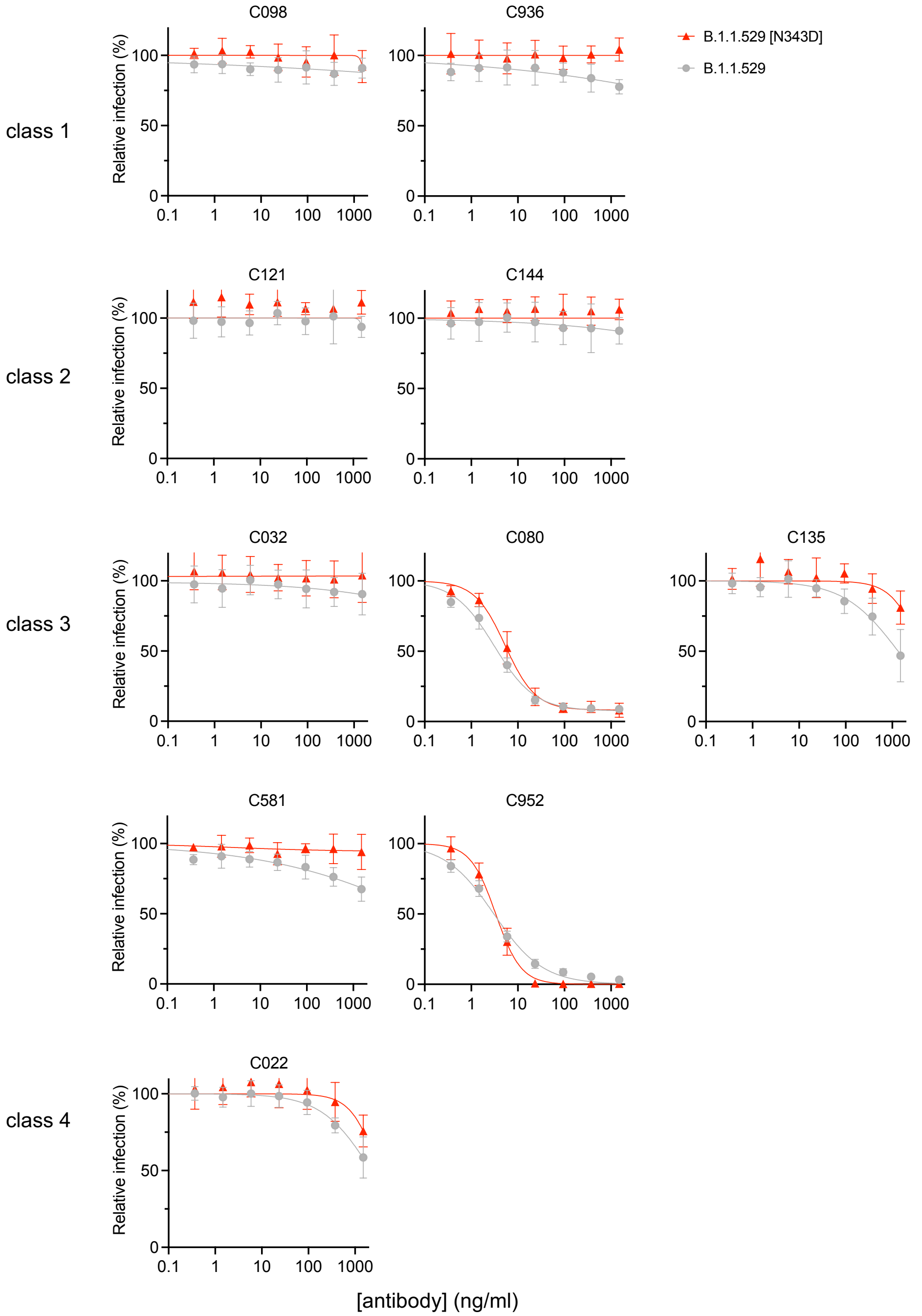

FIG S7 Effect of N343D substitution on in omicron BA.1 (B.1.1.529) neutralization sensitivity

Quantification of neutralization of glycosylation site mutant N343D in the background of furin uncleavable (R683G) omicron (B.1.1.529) S pseudotyped virus infection in the presence of the indicated concentrations of a panel of monoclonal antibodies, including class 1 (C098 and C936), class 2 (C121 and C144), class 3 (C032, C080, C135, C581, and C952), and class 4 (C022) antibodies. The mean and range of two independent experiments (two technical replicates in each experiment) are shown.

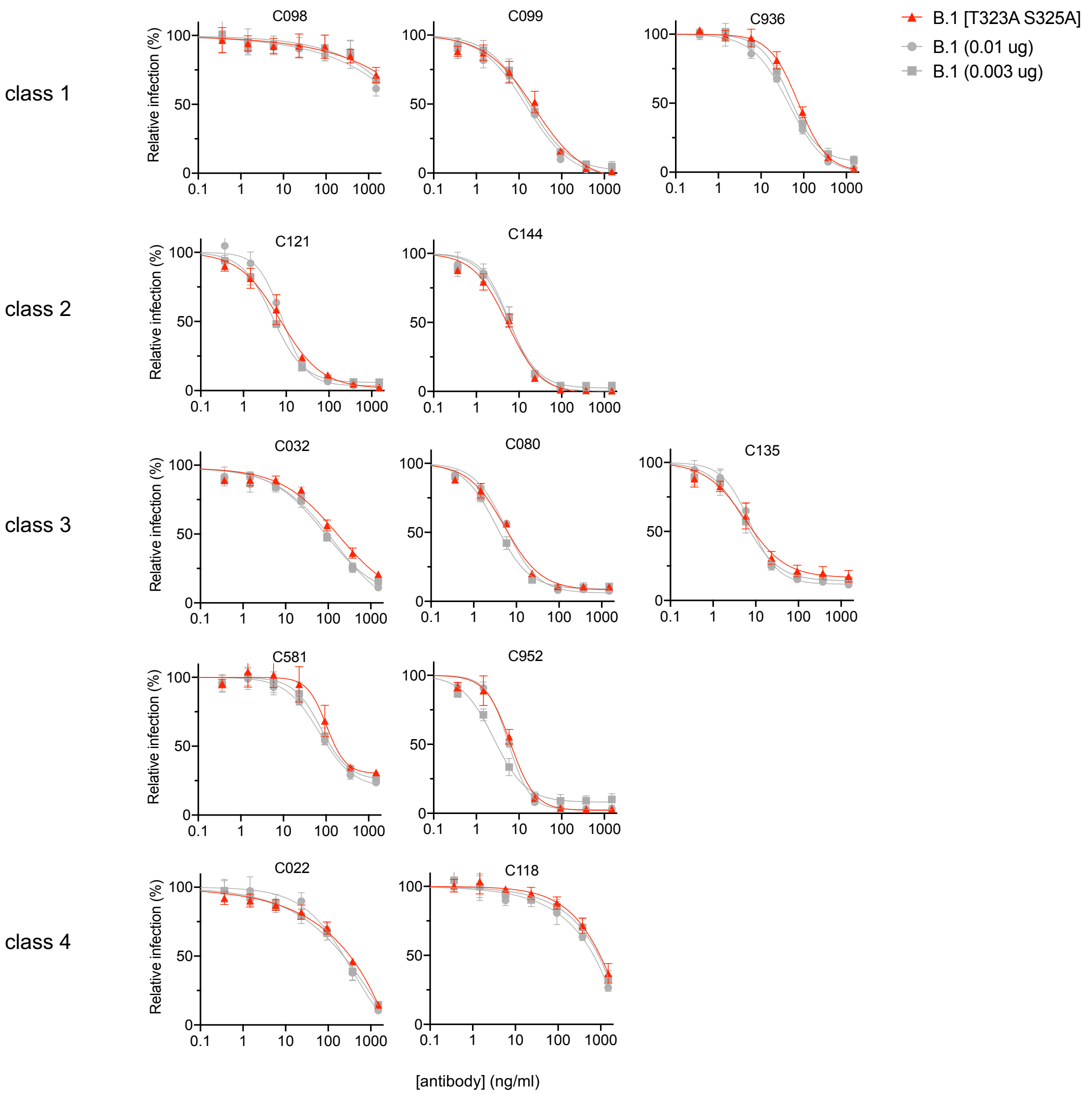

FIG S8 Mutations of the O-linked glycosylation sites at 323 and 325 in the RBD (T323A S325A) have marginal effect on neutralization sensitivity

Neutralization of glycosylation site mutant T323A S325A pseudotyped virus infection in the presence of the indicated concentrations of a panel of monoclonal antibodies, including class 1 (C098, C099, and C936), class 2 (C121 and C144), class 3 (C032, C080, C135, C581, and C952), and class 4 (C022 and C118) antibodies. As controls, glycosylation intact S expression plasmid (WT B.1 in the furin uncleavable R683G background) was transfected at two doses, 10 ng or 3 ng, and the resulting viruses were assessed for neutralization sensitivity in parallel. The mean and range of two independent experiments (two technical replicates in each experiment) are shown.

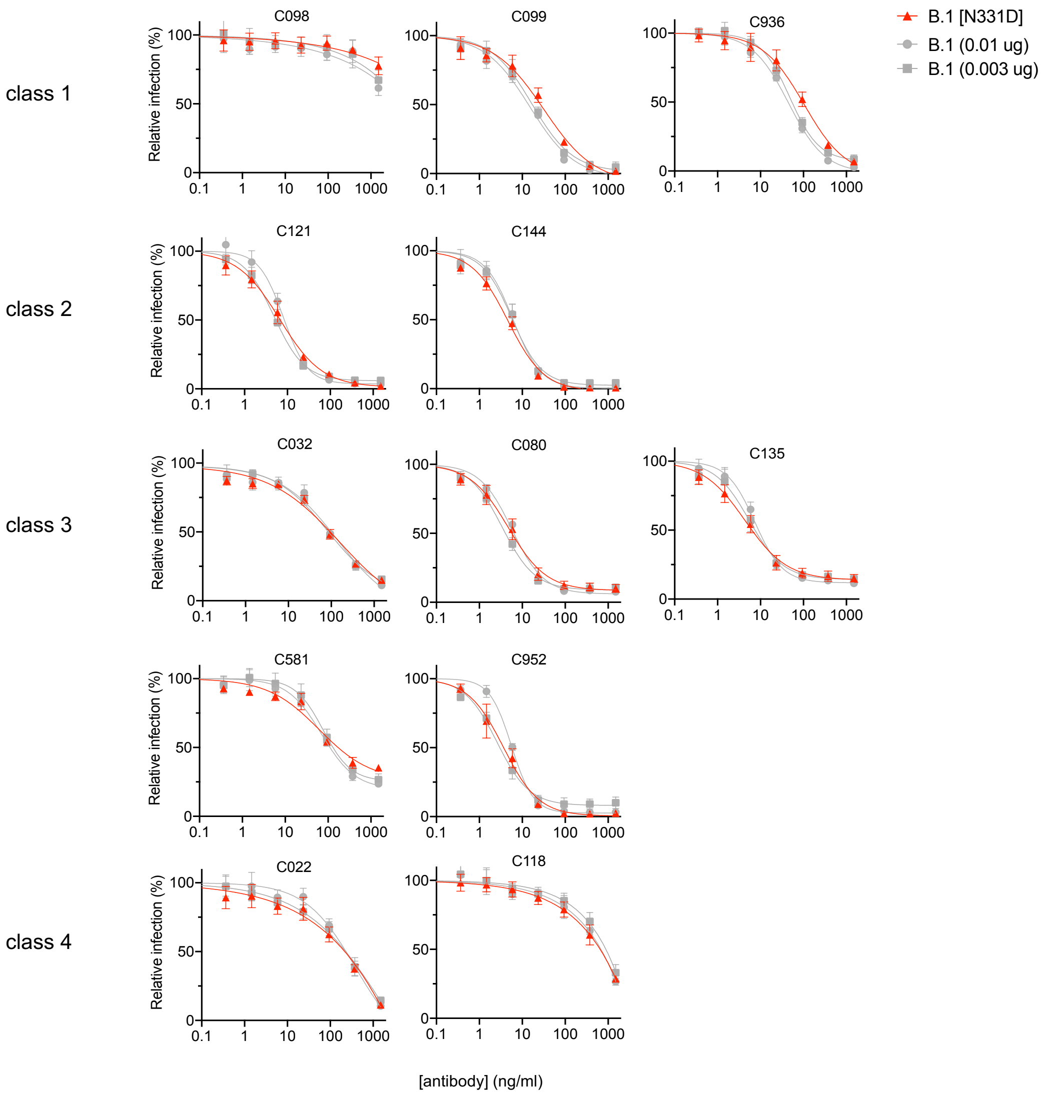

FIG S9 Effect of glycosylation at N331 on neutralization sensitivity
Neutralization of glycosylation site mutant N331D pseudotyped virus infection in the presence of the indicated concentrations of a panel of monoclonal antibodies, including class 1 (C098, C099, and C936), class 2 (C121 and C144), class 3 (C032, C080, C135, C581, and C952), and class 4 (C022 and C118) antibodies. As controls, glycosylation intact S (WT B.1 in the furin uncleavable R683G background) was transfected at two doses, 10 ng or 3 ng, and the resulting viruses were assessed for neutralization sensitivity in parallel. The mean and range of two independent experiments (two technical replicates in each experiment) are shown.

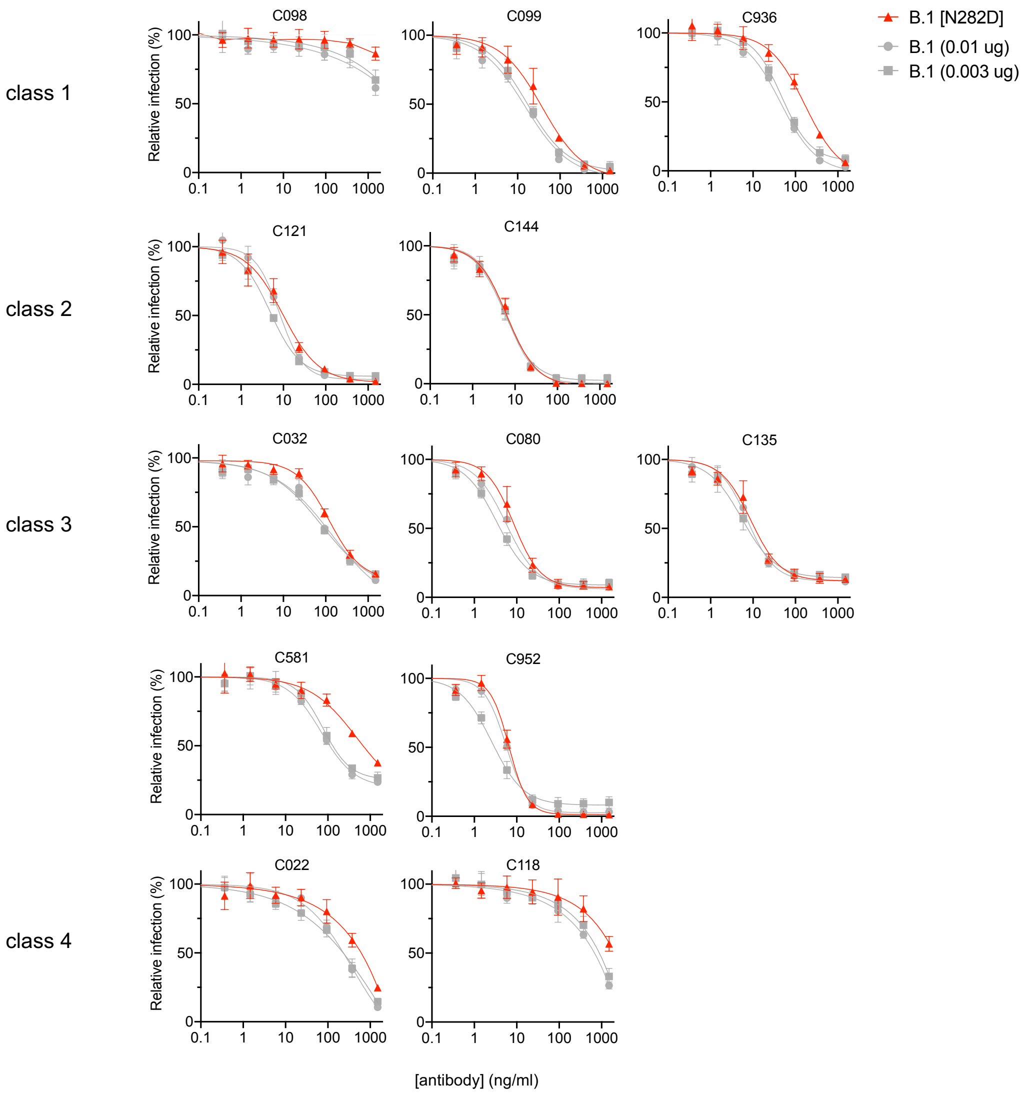

FIG S10 Effect of glycosylation at N282 on neutralization sensitivity
Neutralization of glycosylation site mutant N282D pseudotyped virus infection in the presence of the indicated concentrations of a panel of monoclonal antibodies, including class 1 (C098, C099, and C936), class 2 (C121 and C144), class 3 (C032, C080, C135, C581, and C952), and class 4 (C022 and C118). As controls, glycosylation intact S (WT B.1 in the furin uncleavable R683G background) was transfected at two doses, 10 ng or 3 ng, and the resulting viruses were assessed for neutralization sensitivity in parallel. The mean and range of two independent experiments (two technical replicates in each experiment) are shown.

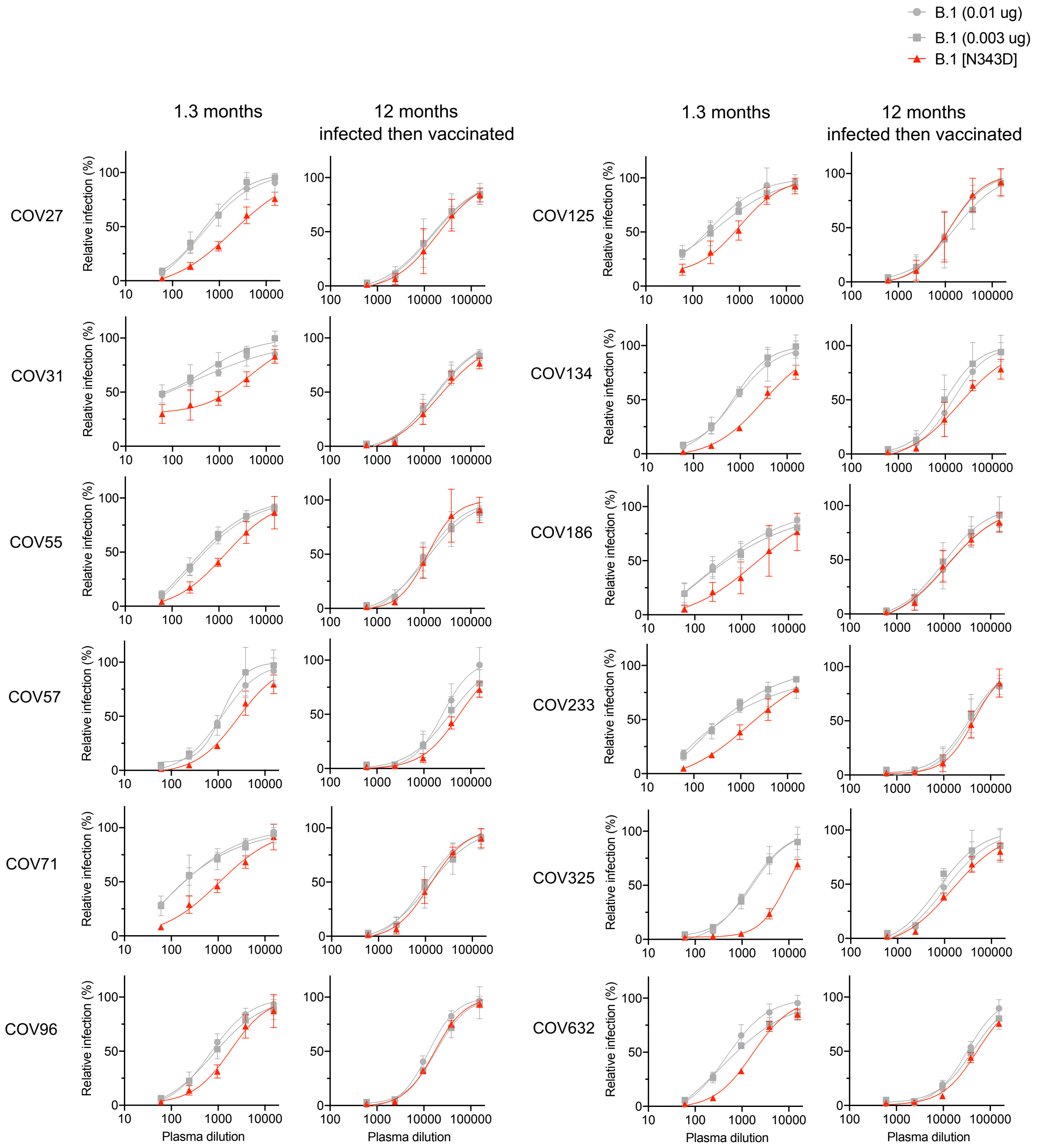

FIG S11 Neutralization sensitivity of N343D mutant to convalescent plasma
Additional examples of plasma neutralization of N343D or glycosylation site intact S (in the furin uncleavable R683G background, same as FIG 5) pseudotyped virus using 293T/ACE2.cl22 target cells. The mean and range of two technical replicates are shown.

Table S1: Infectiousness of HIV-1 pseudotypes bearing SARS-CoV-2 glycosylation site mutant spike proteins

	1st experiment	2nd experiment	Average	STDEV
N17D	6.50E+08	8.93E+08	7.31E+08	1.72E+08
N61D	2.48E+07	2.94E+07	2.63E+07	3.25E+06
N74D	7.71E+08	9.05E+08	8.15E+08	9.51E+07
N122D	3.17E+08	2.74E+08	3.03E+08	3.04E+07
N149D	6.09E+08	5.98E+08	6.05E+08	7.42E+06
N165D	2.03E+08	2.30E+08	2.12E+08	1.94E+07
N234D	6.12E+08	3.52E+08	5.25E+08	1.84E+08
N282D	5.93E+08	4.66E+08	5.51E+08	8.98E+07
T323A S325A	1.59E+08	1.55E+08	1.58E+08	2.83E+06
N331D	2.10E+08	2.67E+08	2.29E+08	4.07E+07
N343D	1.14E+08	6.90E+07	9.90E+07	3.18E+07
N603D	8.47E+08	1.17E+09	9.55E+08	2.28E+08
N616D	3.72E+08	4.68E+08	4.04E+08	6.82E+07
N657D	2.04E+08	2.54E+08	2.21E+08	3.54E+07
N709D	1.55E+09	1.49E+09	1.53E+09	4.24E+07
N717D	2.27E+08	2.51E+08	2.35E+08	1.73E+07
N801D	6.89E+08	8.16E+08	7.31E+08	8.98E+07
N1074D	3.67E+08	4.11E+08	3.81E+08	3.15E+07
N1098D	5.66E+08	7.91E+08	6.41E+08	1.59E+08
N1134D	1.05E+09	9.71E+08	1.02E+09	5.59E+07
N1158D	5.24E+08	5.43E+08	5.30E+08	1.34E+07
N1173D	6.43E+08	6.01E+08	6.29E+08	2.97E+07
N1194D	8.39E+07	9.27E+07	8.68E+07	6.22E+06
B.1	1.17E+09	1.14E+09	1.16E+09	1.77E+07

Infectious titers in relative light units (RLU) per ml of HIV-1 pseudotypes containing an packaged NanoLuc luciferase expression vector and bearing wild type or glycosylation site mutant SARS-CoV-2 spike proteins.
